# Supplementary material for: A new allele of acid soil tolerance gene from a malting barley variety
Source: BMC Genet. 2015 Jul 29;16:92. doi: 10.1186/s12863-015-0254-4 (PMC4518660; doi:10.1186/s12863-015-0254-4)
Supplement: Additional file 1: — Segregation distortion of markers in the region of Al tolerance gene on 4H; * means significance at 0.05, ** means significance at 0.01. (DOCX 34 kb) [file 12863_2015_254_MOESM1_ESM.docx]

Additional file 1

| Marker | BR2 | Hamelin | Missing | χ^2^ | Significance. |
| --- | --- | --- | --- | --- | --- |
| HVM03 | 86 | 53 | 19 | 7.83 | ** |
| Bmag353 | 103 | 49 | 6 | 19.18 | ** |
| Bmac310 | 84 | 48 | 26 | 9.82 | ** |
| Bmac186 | 101 | 42 | 15 | 24.34 | ** |
| bmag740 | 88 | 55 | 15 | 7.62 | ** |
| Ebmac775 | 83 | 54 | 21 | 6.14 | * |
| Cit7 | 104 | 54 | 0 | 15.82 | ** |
