# Supplementary figures and images for: A new allele of acid soil tolerance gene from a malting barley variety
Source: BMC Genet. 2015 Jul 29;16:92. doi: 10.1186/s12863-015-0254-4 (PMC4518660; doi:10.1186/s12863-015-0254-4)

## Slide 1
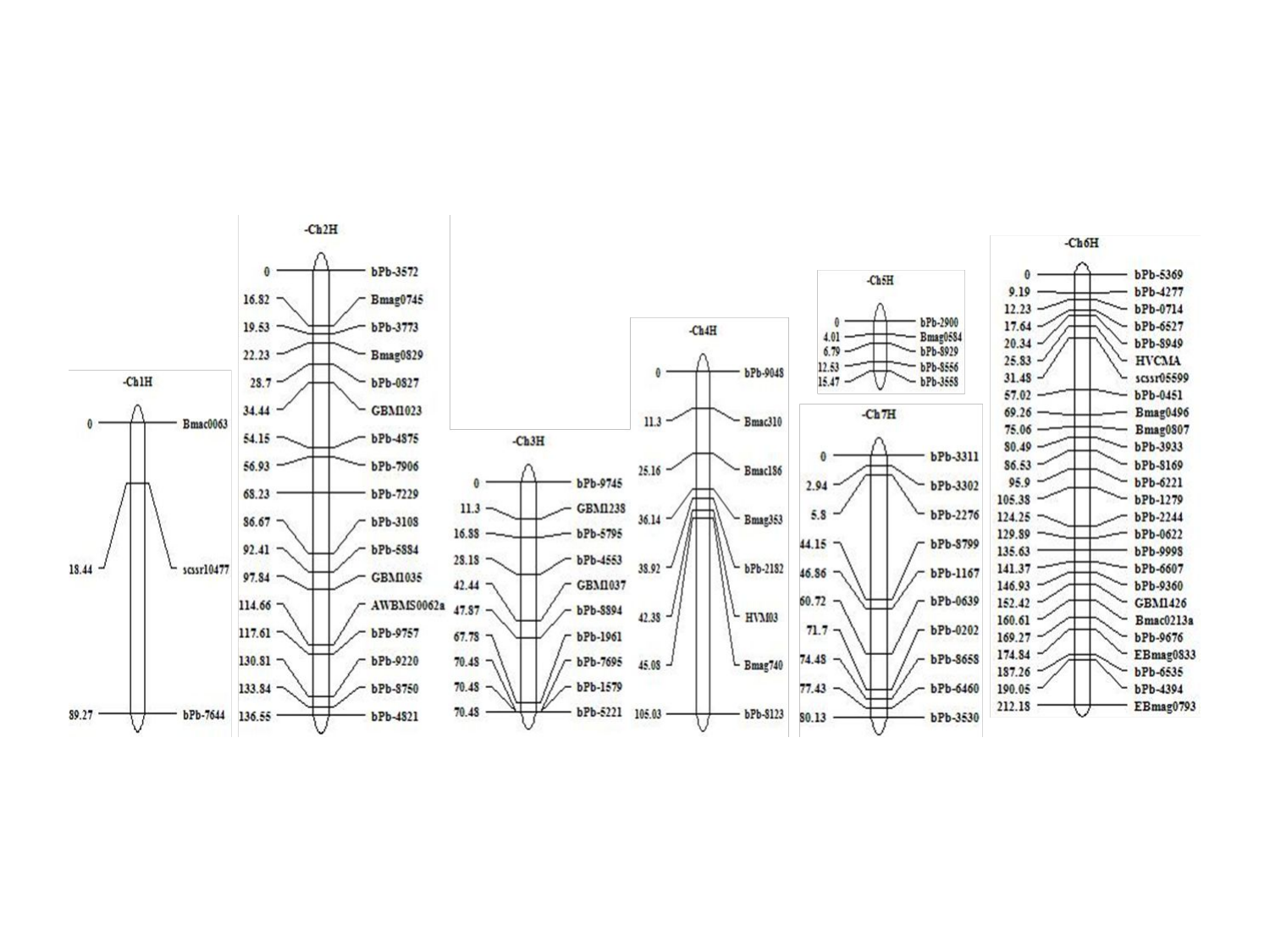

Supplement: Additional file 2: — Linkage map of DArT and SSR markers in Hamelin/Br2 DH population. (PPTX 814 kb) [file 12863_2015_254_MOESM2_ESM.pptx]

## Slide 1
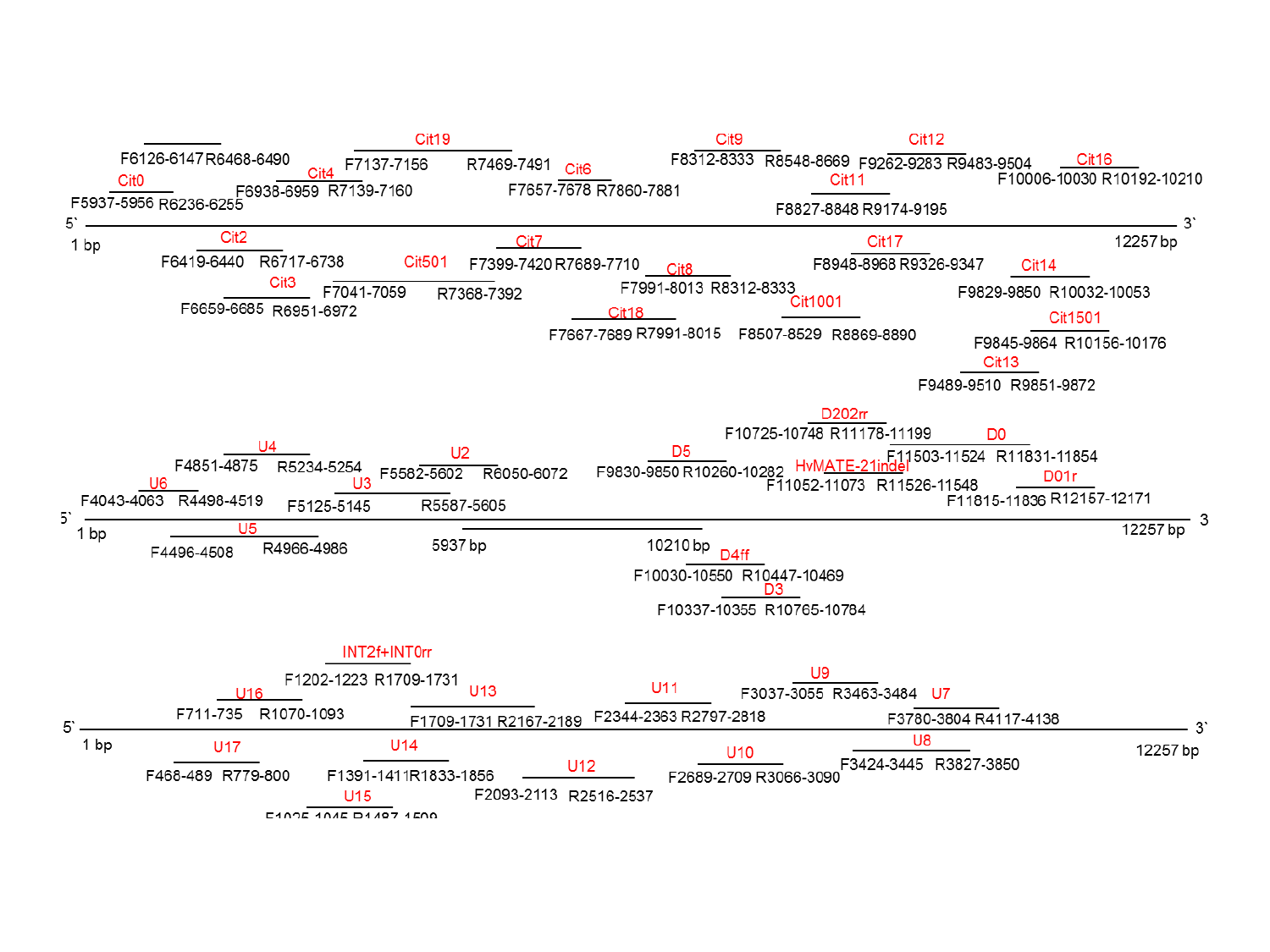

Supplement: Additional file 3: — Primers’ position and coverage of HvMATE gene (Primers’ names are highlighted in red). F: forward primer position (bp), R: reverse primer position (bp). (PPTX 369 kb) [file 12863_2015_254_MOESM3_ESM.pptx]
