# Supplementary material for: A new allele of acid soil tolerance gene from a malting barley variety
Source: BMC Genet. 2015 Jul 29;16:92. doi: 10.1186/s12863-015-0254-4 (PMC4518660; doi:10.1186/s12863-015-0254-4)
Supplement: Additional file 4: — SNP and INDEL of HvMATE gene in 6 barley cultivars. (DOCX 48 kb) [file 12863_2015_254_MOESM4_ESM.docx]

**Additional file 4**

| Whole sequence  Position | SNP/indel | Amino acid change | mRNA  change | Haruna Nijo | Br2 | Hamelin | Svanhals | Morex | Murasakimochi | Inserted Sequence |
| --- | --- | --- | --- | --- | --- | --- | --- | --- | --- | --- |
| 2319 | SNP | No | No | - | T | G | T | G | G | - |
| 2331 | SNP | No | No | - | G | A | G | A | A | - |
| 2724 | SNP | No | No | - | T | G | T | G | G | - |
| 3189 | SNP | No | No | - | C | T | C | T | T | - |
| 3197-4219 | indel | No | No | - | deletion | deletion | deletion | deletion | insertion | 1, 023 kb |
| 4398 | SNP | No | No | - | T | C | T | C | C | - |
| 4470 | indel | No | No | - | deletion | insertion | deletion | insertion | insertion | 1 bp |
| 4537 | SNP | No | No | - | C | G | C | G | G | - |
| 4659 | SNP | No | No | - | G | G | C | G | G | - |
| 4891 | SNP | No | No | - | A | G | A | G | G | - |
| 4896 | SNP | No | No | - | A | G | A | G | G | - |
| 4912 | SNP | No | No | - | T | C | T | C | C | - |
| 4968 | SNP | No | No | - | T | G | T | G | G | - |
| 5025 | SNP | No | No | - | T | C | T | C | C |  |
| 5066 | SNP | No | No | - | C | C | C | C | T | - |
| 5140-5169 | indel | No | No | - | deletion | insertion | deletion | insertion | insertion | 29 bp |
| 5212 | SNP | No | No | - | T | G | T | G | G | - |
| 5220 | SNP | No | No | - | G | A | G | A | A | - |
| 5364 | SNP | No | No | - | A | G | A | G | G | - |
| 5374-5379 | indel | No | No | - | insertion | deletion | insertion | deletion | deletion | 6 bp |
| 5479 | indel | No | No | - | deletion | insertion | deletion | insertion | insertion | 1 bp |
| 5509 | SNP | No | No | - | C | T | C | T | T | - |
| 5941 | SNP | No | No | - | C | T | C | T | T | - |
| 6051 | SNP | No | No | - | C | A | C | A | A | - |
| 6278 | indel | No | No | - | insertion | deletion | insertion | deletion | deletion | 1 bp |
| 6357 | SNP | No | No | - | T | C | T | C | C | - |
| 6470 | SNP | No | No | - | T | C | T | C | C | - |
| 6551 | SNP | No | No | - | C | T | C | T | T | - |
| 6570 | SNP | No | No | - | G | G | G | G | A | - |
| 6690 | SNP | No | No | - | A | G | A | G | G | - |
| 6758 | indel | No | No | - | deletion | insertion | deletion | insertion | insertion | 1 bp |
| 6806 | SNP | No | No | - | C | T | C | T | C |  |
| 6989 | SNP | No | No | - | G | A | G | A | G |  |
| 7341 | SNP | No | No | - | G | C | G | C | C |  |
| 7361 | SNP | No | No | - | C | T | C | T | T |  |
| 7400 | SNP | No | No | - | C | T | C | T | T |  |
| 7794 | SNP | No | No | - | T | G | T | G | G |  |
| 7840 | SNP | No | No | - | T | C | T | C | T |  |
| 7883 | SNP | No | No | - | G | A | G | A | G |  |

**Continued**

| Whole sequence  Position | SNP/indel | Amino acid change | mRNA  change | Haruna Nijo | Br2 | Hamelin | Svanhals | Morex | Murasakimochi | Inserted Sequence |
| --- | --- | --- | --- | --- | --- | --- | --- | --- | --- | --- |
| 8801 | SNP | No | No | C | G | C | G | C | G |  |
| 10168 | SNP | Yes | Yes | T(L) | G (V) | T (L) | G (V) | T (L) | G (V) |  |
| 10648 | SNP | Yes | Yes | C(P) | C (P) | C (P) | C (P) | C (P) | A (H) |  |
| 10711 | SNP | No | Yes | C | C | C | C | C | T |  |
| 11253 | SNP | No | Yes | G | G | G | A | G | A |  |
| 12175 | indel | No | No | deletion | deletion | deletion | deletion | deletion | insertion | 1 bp |
| 12619-12620 | indel | No | Yes | deletion | insertion | deletion | insertion | - | - | 2 bp |
| 12660 | SNP | No | Yes | G | A | G | A | - | - |  |
| 12739 | SNP | No | No | T | C | T | C | - | - |  |
| 12765 | SNP | No | No | A | G | A | G | - | - |  |
| 12775 | SNP | No | No | G | C | G | C | - | - |  |
| 12793 | SNP | No | No | G | A | G | A | - | - |  |
| 12831 | indel | No | No | deletion | insertion | deletion | insertion | - | - | 1 bp |
| 12832 | SNP | No | No | A | G | A | G | - | - |  |
| 12851 | indel | No | No | deletion | insertion | deletion | insertion | - | - | 1 bp |
| 12885 | SNP | No | No | C | A | C | A | - | - | - |
| 12901 | SNP | No | No | T | C | T | C | - | - | - |
| 12937 | SNP | No | No | - | C | C | T | - | - | - |
| 12959 | SNP | No | No | - | G | A | G | - | - | - |
| 13122 | SNP | No | No | - | A | T | A | - | - | - |
| 13175 | SNP | No | No | - | A | G | A | - | - | - |
| 13191 | SNP | No | No | - | A | G | A | - | - | - |
| 13222 | SNP | No | No | - | G | A | G | - | - | - |
| 13252 | SNP | No | No | - | T | C | C | - | - | - |
| 13301 | SNP | No | No | - | A | G | A | - | - | - |
| 13320 | SNP | No | No | - | T | C | T | - | - | - |
| 13344 | SNP | No | No | - | C | T | C | - | - | - |
| 13367 | SNP | No | No | - | A | G | A | - | - | - |
| 13389 | SNP | No | No | - | C | G | C | - | - | - |
| 13425 | SNP | No | No | - | A | G | A | - | - | - |
| 13456 | SNP | No | No | - | G | A | G | - | - | - |
| 13519 | SNP | No | No | - | T | C | T | - | - | - |
| 13530 | SNP | No | No | - | C | A | C | - | - | - |
| 13621 | SNP | No | No | - | T | C | C | - | - | - |
| 13622 | SNP | No | No | - | G | A | G | - | - | - |
| 13636 | SNP | No | No | - | C | G | G | - | - | - |
| 13771 | SNP | No | No | - | A | G | A | - | - | - |
| 13819 | SNP | No | No | - | G | A | G | - | - | - |

**Continued**

| Whole sequence  Position | SNP/indel | Amino acid change | mRNA  change | Haruna Nijo | Br2 | Hamelin | Svanhals | Morex | Murasakimochi | Inserted Sequence |
| --- | --- | --- | --- | --- | --- | --- | --- | --- | --- | --- |
| 13840 | SNP | No | No | - | G | C | G | - | - | - |
| 13878 | SNP | No | No | - | C | T | C | - | - | - |
| 14000 | SNP | No | No | - | G | C | G | - | - | - |
| 14112 | SNP | No | No | - | A | G | A | - | - | - |
| 14118 | SNP | No | No | - | G | A | G | - | - | - |
| 14121 | SNP | No | No | - | A | G | A | - | - | - |
| 14148 | SNP | No | No | - | C | A | C | - | - | - |
| 14153 | SNP | No | No | - | C | G | G | - | - | - |
| 14164-14184 | indel | No | No | - | deletion | insertion | deletion | - | - | 21bp |
| 14227 | SNP | No | No | - | A | G | G | - | - | - |
| 14272 | SNP | No | No | - | T | C | C | - | - | - |
| 14274 | SNP | No | No | - | T | C | T | - | - | - |
| 14301 | SNP | No | No | - | T | C | T | - | - | - |
| 14308 | SNP | No | No | - | C | T | C | - | - | - |
| 14477 | SNP | No | No | - | G | A | G | - | - | - |
| 14497 | SNP | No | No | - | C | A | C | - | - | - |
| 14515-14541 | indel | No | No | - | deletion | insertion | deletion | - | - | 29bp |
| 14565 | indel | No | No | - | insertion | deletion | insertion | - | - | 1 bp |
| 14650 | SNP | No | No | - | G | T | G | - | - | - |
| 14660 | SNP | No | No | - | G | A | G | - | - | - |
| 14700 | SNP | No | No | - | C | T | C | - | - | - |
| 14773 | SNP | No | No | - | C | T | C | - | - | - |
| 14855 | SNP | No | No | - | C | T | C | - | - | - |
