# Supplementary material for: A new allele of acid soil tolerance gene from a malting barley variety
Source: BMC Genet. 2015 Jul 29;16:92. doi: 10.1186/s12863-015-0254-4 (PMC4518660; doi:10.1186/s12863-015-0254-4)
Supplement: Additional file 5: — Fifty-six barley accessions and their origins. (DOCX 37 kb) [file 12863_2015_254_MOESM5_ESM.docx]

**Additional file 5**

| **Accession names** | **Origin** |  | **Accession names** | **Origin** |  | **Accession names** | **Origin** |  |  |
| --- | --- | --- | --- | --- | --- | --- | --- | --- | --- |
| Svanhals | CYMMIT |  | Spanish Landrace-316 | Spain |  | Macquarie | Australia |  |  |
| Cavmen | Australia |  | Spanish Landrace-333c | Spain |  | TF026 | Australia |  |  |
| Hindmarsh | Australia |  | Portuguese Landrace | Portugal |  | YF374 | China |  |  |
| Buloke | Australia |  | MAR-86-E1138 | Unknown |  | Tx9425 | China |  |  |
| Baudin | Australia |  | Spanish Landrace-336d | Spain |  | Unicorn | Japan |  |  |
| Cevada de 6 Ordens | Portugal |  | Spanish Landrace-338c | Spain |  | Xiaojiang | China |  |  |
| Cevada Preta | Portugal |  | Vlamingh | Australia |  | Yan90260 | China |  |  |
| Spanish Landrace-352 | Spain |  | HOR13461 | Spain |  | YUQS | China |  |  |
| Spanish Landrace-355 | Spain |  | Cevada de 2 Ordens | Portugal |  | YWHKSL | China |  |  |
| Noire Maroc | Morocco |  | Spanish Landrace-349 | Spain |  | Yiwu Erleng | China |  |  |
| Precoce du Maroc | Morocco |  | Spanish Landrace-349b | Spain |  | Kinu nijo 6 | Japan |  |  |
| Baudin | Australia |  | 93-3143 | Unknown |  | YYXT | China |  |  |
| Boa Fe | Portugal |  | Lixi 143 | China |  | Zhepi 2 | China |  |  |
| Barlis | Morocco |  | ZUG293 | China |  | YPSLDM | China |  |  |
| Keka | Spain |  | ZUG403 | China |  | Macquarie | Australia |  |  |
| Rosa | Unknown |  | YU6472 | China |  | Macquarie | Australia |  |  |
| Gairdner | Australia |  | Brindabella | Australia |  |  |  |  |  |
| Moroccan Landrace | Morocco |  | Numar | America |  |  |  |  |  |
| Spanish-309d | Spain |  | Dash | New Zealand |  |  |  |  |  |
| HOR12517 | Spain |  | Dayton | Australia |  |  |  |  |  |
